# Supplementary material for: Soluble CD27 differentially predicts resistance to anti-PD1 alone but not with anti-CTLA-4 in melanoma
Source: EMBO Mol Med. 2025 Mar 27;17(5):909–22. doi: 10.1038/s44321-025-00203-9 (PMC12081602; doi:10.1038/s44321-025-00203-9)
Supplement: Supplementary file 1 — Table EV1 [file 44321_2025_203_MOESM1_ESM.docx]

| **PREDIMEL** |  | **OS** | | | | | **PFS** | | | | |
| --- | --- | --- | --- | --- | --- | --- | --- | --- | --- | --- | --- |
| **Parameters** | **Values** | **N** | **N event** | **HR** | **95% CI** | **P value** | **N** | **N event** | **HR** | **95% Cl** | **P value** |
| **Age >= 75** | No | 74 | 6 | 1·00 |  |  | 74 | 37 | 1·00 |  |  |
|  | Yes | 8 | 1 | 1·85 | (0·22-15·58) | 0·57 | 8 | 7 | 2·39 | (1·05-5·4) | 0·037 |
| **Sex** | Male | 54 | 5 | 1·00 |  |  | 54 | 26 | 1·00 |  |  |
|  | Female | 28 | 2 | 0·83 | (0·16-4·26) | 0·82 | 28 | 18 | 1·62 | (0·89-2·97) | 0·12 |
| **Stade AJCC M1c** | No | 39 | 4 | 1·00 |  |  | 39 | 20 | 1·00 |  |  |
|  | Yes | 43 | 3 | 0·73 | (0·16-3·27) | 0·68 | 43 | 24 | 1·19 | (0·65-2·15) | 0·57 |
| **Braf mutation** | No | 68 | 6 | 1·00 |  |  | 68 | 37 | 1·00 |  |  |
|  | Yes | 14 | 1 | 0·78 | (0·09-6·49) | 0·82 | 14 | 7 | 0·95 | (0·42-2·14) | 0·91 |
| **Nras mutation** | No | 70 | 5 | 1·00 |  |  | 70 | 35 | 1·00 |  |  |
|  | Yes | 12 | 2 | 2·37 | (0·46-12·2) | 0·30 | 12 | 9 | 2·01 | (0·96-4·2) | 0·063 |
| **ECOG PS** | >= 2 | 18 | 0 |  |  |  | 18 | 15 | 1·00 |  |  |
|  | 0 or 1 | 53 | 3 |  |  |  | 53 | 24 | 0·36 | (0·19-0·7) | 0·002 |
| **LDH/100** |  | 75 | 6 | 1·1 | (0·96-1·26) | 0·17 | 75 | 41 | 1·1 | (1·03-1·16) | 0·002 |
| **Neutrophils/Lymphocytes** |  | 78 | 5 | 1·06 | (0·73-1·53) | 0·75 | 78 | 41 | 1·18 | (1·04-1·35) | 0·009 |
| **PD-L1** | No | 21 | 3 | 1·00 |  |  | 21 | 13 | 1·00 |  |  |
|  | Yes | 13 | 1 | 0·55 | (0·06-5·28) | 0·60 | 13 | 3 | 0·28 | (0·08-1) | 0·050 |
| **Brain metastasis** | No | 77 | 7 |  |  |  | 77 | 42 | 1·00 |  |  |
|  | Yes | 5 | 0 |  |  |  | 5 | 2 | 0·56 | (0·14-2·32) | 0·42 |
| **Liver metastasis** | No | 63 | 7 |  |  |  | 63 | 34 | 1·00 |  |  |
|  | Yes | 19 | 0 |  |  |  | 19 | 10 | 1·09 | (0·54-2·21) | 0·81 |
| **sCD27** |  | 64 | 5 | 1·02 | (0·83-1·25) | 0·84 | 64 | 33 | 1·03 | (0·95-1·12) | 0·47 |
| **sCD27 >100 U/ml** | No | 43 | 3 | 1·00 |  |  | 43 | 21 | 1·00 |  |  |
|  | Yes | 21 | 2 | 1·53 | (0·26-9·17) | 0·64 | 21 | 12 | 1·37 | (0·67-2·78) | 0·39 |
| **TMB** |  | 27 | 2 | 1.00 | (0.95 - 1.04) | 0.830 | 27 | 14 | 0.98 | (0.95 - 1.02) | 0.31 |
| **TMB>10 mut/Mb** | No | 15 | 1 | 1.00 |  |  | 15 | 10 | 1 |  |  |
|  | Yes | 12 | 1 | 1.00 | (0.06 - 15.97) | 1.000 | 12 | 4 | 0.37 | (0.12 - 1.18) | 0.094 |
| **Total CD8 cells/mm2** |  | 23 | 2 | 1.00 | (0.99 - 1) | 0.600 | 23 | 12 | 1 | (1 - 1) | 0.085 |
| **CRP** |  | 64 | 5 | 1.01 | (1 - 1.02) | 0.002 | 64 | 33 | 1.01 | (1 - 1.01) | 0.007 |
| **CRP>5 mg/mL** | No | 30 | 2 | 1.00 |  |  | 30 | 13 | 1 |  |  |
|  | Yes | 34 | 3 | 1.41 | (0.24 - 8.47) | 0.700 | 34 | 20 | 1.66 | (0.82 - 3.33) | 0.16 |
| **IL-6>10 pg/mL** | No | 53 | 2 | 1.00 |  |  | 53 | 25 | 1 |  |  |
|  | Yes | 11 | 3 | 9.14 | (1.52 - 54.91) | 0.016 | 11 | 8 | 2.49 | (1.12 - 5.54) | 0.025 |

**Table EV1.** **Clinical and biological variables in the prediction of OS and PFS in the Predimel cohort of melanoma patients treated by the combination therapy**

The univariate Cox’s model Hazard Ratios (HRs) for OS and PFS and 95% confidence intervals of baseline clinical and biological variables. Concentration of sCD27 were either evaluated as a continuous variable or dichomized on the 100U/ml cut-off. Two-sided p < 0.05 was considered significant.
