# Supplementary material for: Soluble CD27 differentially predicts resistance to anti-PD1 alone but not with anti-CTLA-4 in melanoma
Source: EMBO Mol Med. 2025 Mar 27;17(5):909–22. doi: 10.1038/s44321-025-00203-9 (PMC12081602; doi:10.1038/s44321-025-00203-9)
Supplement: Supplementary file 2 — Table EV2 [file 44321_2025_203_MOESM2_ESM.docx]

**Table EV2 : Predictive factors of complete clinical responses (CR) at 12 months in the group of patients treated with anti-PD-1 in the 2 Predimel and Melbase cohorts.** Biological and clinical variables considered as a continuous (age, PNN/lymphocyte ratio, LDH) or dichotomized (sex, Braf mutation, Nras mutation, cerebral metastasis, more than 3 metastatic sites, ECOG, LDH > normal values, AJCC M1c ) at inclusion in the Predimel or Melbase cohorts in patients treated with anti-PD-1 alone
