# Supplementary material for: Soluble CD27 differentially predicts resistance to anti-PD1 alone but not with anti-CTLA-4 in melanoma
Source: EMBO Mol Med. 2025 Mar 27;17(5):909–22. doi: 10.1038/s44321-025-00203-9 (PMC12081602; doi:10.1038/s44321-025-00203-9)
Supplement: Supplementary file 3 — Table EV3 [file 44321_2025_203_MOESM3_ESM.docx]

| **MelBase** |  | **OS** | | | | | **PFS** | | | | |
| --- | --- | --- | --- | --- | --- | --- | --- | --- | --- | --- | --- |
| **Parameters** | **Values** | **N** | **N event** | **HR** | **95% CI** | **P value** | **N** | **N event** | **HR** | **95% CI** | **P value** |
| **Age >= 75** | No | 102 | 42 | 1·00 |  |  | 102 | 62 | 1·00 |  |  |
|  | Yes | 6 | 4 | 1·77 | (0·63-4·96) | 0·28 | 6 | 4 | 0·93 | (0·34-2·56) | 0·89 |
| **Sex** | Male | 63 | 26 | 1·00 |  |  | 63 | 39 | 1·00 |  |  |
|  | Female | 45 | 20 | 1·08 | (0·6-1·94) | 0·80 | 45 | 27 | 0·96 | (0·59-1·56) | 0·86 |
| **ECOG>=2** | >=2 | 26 | 18 | 1·00 |  |  | 26 | 19 | 1·00 |  |  |
|  | 0 or 1 | 82 | 28 | 0·26 | (0·14-0·48) | <0·0001 | 82 | 47 | 0·43 | (0·25-0·73) | 0·002 |
| **Stade AJCC M1c** | No | 48 | 20 | 1·00 |  |  | 48 | 29 | 1·00 |  |  |
|  | Yes | 60 | 26 | 1·34 | (0·74-2·42) | 0·33 | 60 | 37 | 1·21 | (0·74-1·97) | 0·44 |
| **Braf mutation** | No | 62 | 32 | 1·00 |  |  | 62 | 40 | 1·00 |  |  |
|  | Yes | 45 | 14 | 0·47 | (0·25-0·88) | 0·018 | 45 | 26 | 0·73 | (0·45-1·2) | 0·22 |
| **Nras mutation** | No | 60 | 28 | 1·00 |  |  | 60 | 39 | 1·00 |  |  |
|  | Yes | 30 | 12 | 0·79 | (0·39-1·59) | 0·52 | 30 | 16 | 0·77 | (0·43-1·37) | 0·37 |
| **Brain metastasis** | No | 86 | 36 | 1·00 |  |  | 86 | 53 | 1·00 |  |  |
|  | Yes | 20 | 10 | 1·57 | (0·77-3·17) | 0·21 | 20 | 12 | 1·27 | (0·68-2·37) | 0·46 |
| **Liver metastasis** | No | 79 | 33 | 1·00 |  |  | 79 | 47 | 1·00 |  |  |
|  | Yes | 27 | 13 | 1·31 | (0·69-2·5) | 0·41 | 27 | 18 | 1·23 | (0·72-2·12) | 0·45 |
| **LDH/100** |  | 99 | 42 | 1·09 | (1·06-1·13) | <0·0001 | 99 | 61 | 1·04 | (1·01-1·06) | 0·002 |
| **Neutrophils/Lymphocytes** |  | 104 | 44 | 1·04 | (0·95-1·14) | 0·45 | 104 | 64 | 1·01 | (0·92-1·09) | 0·91 |
| **sCD27** |  | 108 | 46 | 1·05 | (1-1·11) | 0·060 | 108 | 66 | 1·03 | (0·98-1·07) | 0·29 |
| **sCD27 > 100 U/ml** | No | 50 | 20 | 1·00 |  |  | 50 | 31 | 1·00 |  |  |
|  | Yes | 58 | 26 | 1·11 | (0·62-2·01) | 0·72 | 58 | 35 | 0·89 | (0·55-1·45) | 0·64 |
| **CRP** |  | 100 | 44 | 1.01 | (1 - 1.01) | 0.002 | 100 | 61 | 1.01 | (1 - 1.01) | 0.019 |
| **CRP>5 mg/mL** | No | 55 | 16 | 1.00 |  |  | 55 | 26 | 1 |  |  |
|  | Yes | 45 | 28 | 2.93 | (1.58 - 5.44) | 0.0006 | 45 | 35 | 2.35 | (1.41 - 3.93) | 0.001 |
| **IL-6>10 pg/mL** | No | 91 | 37 | 1.00 |  |  | 91 | 53 | 1 |  |  |
|  | Yes | 8 | 6 | 3.96 | (1.65 - 9.47) | 0.002 | 8 | 7 | 2.85 | (1.29 - 6.3) | 0.01 |

**Table EV3. Clinical and biological variables in the prediction of OS and PFS in the Melbase cohort of melanoma patients treated by the combination therapy**

The univariate Cox’s model Hazard Ratios (HRs) for OS and PFS and 95% confidence intervals of baseline clinical and biological variables. Concentration of sCD27 were either evaluated as a continuous variable or dichomized on the 100U/ml cut-off. Two-sided p < 0.05 was considered significant.
