# Supplementary material for: Soluble CD27 differentially predicts resistance to anti-PD1 alone but not with anti-CTLA-4 in melanoma
Source: EMBO Mol Med. 2025 Mar 27;17(5):909–22. doi: 10.1038/s44321-025-00203-9 (PMC12081602; doi:10.1038/s44321-025-00203-9)
Supplement: Supplementary file 4 — Table EV4 [file 44321_2025_203_MOESM4_ESM.docx]

| **Overall Survival (N = 210, No. Deaths = 107)** | | | |
| --- | --- | --- | --- |
| Parameters | HR | 95%CI | P value |
| Combination therapy | 1.57 | (0.74 ; 3.31) | 0.24 |
| sCD27>100U/mL | 4.47 | (2.28 ; 8.78) | <0.0001 |
| Interaction | 0.27 | (0.10 ; 0.69) | 0.006 |
| **Progression Free Survival (N = 210, No. Events = 146)** | | | |
| Parameters | HR | 95%CI | P value |
| Combination therapy | 0.89 | (0.54 ; 1.22) | 0.66 |
| sCD27>100U/mL | 1.74 | (1.05 ; 2.87) | 0.032 |
| Interaction | 0.55 | (0.27 ; 1.10) | 0.092 |
| **Clinical Benefit (CR, PR, SD) (N = 179, No. ORR = 120)** | | | |
| Parameters | OR | 95%CI | P value |
| Combination therapy | 0.85 | (0.29 ; 2.48) | 0.77 |
| sCD27>100U/mL | 0.22 | (0.08 ; 0.64) | 0.006 |
| Interaction | 7.01 | (1.62 ; 30.22) | 0.010 |

**Table EV4 : Propensity score to define the differential value or sCD27 to predict the clinical response to monotherapy (anti-PD-1) and combined therapy**

Association between treatment (anti-PD1 alone versus anti-PD-1 and anti-CTLA-4), sCD27 and overall survival (OS), progression-free survival (PFS) and clinical benefit (CR+PR+SD), using an inverse-probability-weighting propensity score approach, in Cox (OS, PFS) and logistic (clinical benefit) regression models in the MelBase cohort. HR=hazards ratio, 95%CI=95% confidence interval, OR=Odds ratio, Combi= treatment with anti-PD-1 and anti-CTLA-4 (reference anti-PD-1).
