# Supplementary material for: Soluble CD27 differentially predicts resistance to anti-PD1 alone but not with anti-CTLA-4 in melanoma
Source: EMBO Mol Med. 2025 Mar 27;17(5):909–22. doi: 10.1038/s44321-025-00203-9 (PMC12081602; doi:10.1038/s44321-025-00203-9)
Supplement: Supplementary file 5 — Appendix [file 44321_2025_203_MOESM5_ESM.pdf]

APPENDIX

**Table of content Appendix**	**Page 1**
**Figures S1 and S2**	**Page 2-4**
**Appendix Tables S1, S2, S3 and S4**	**Page 5-10**

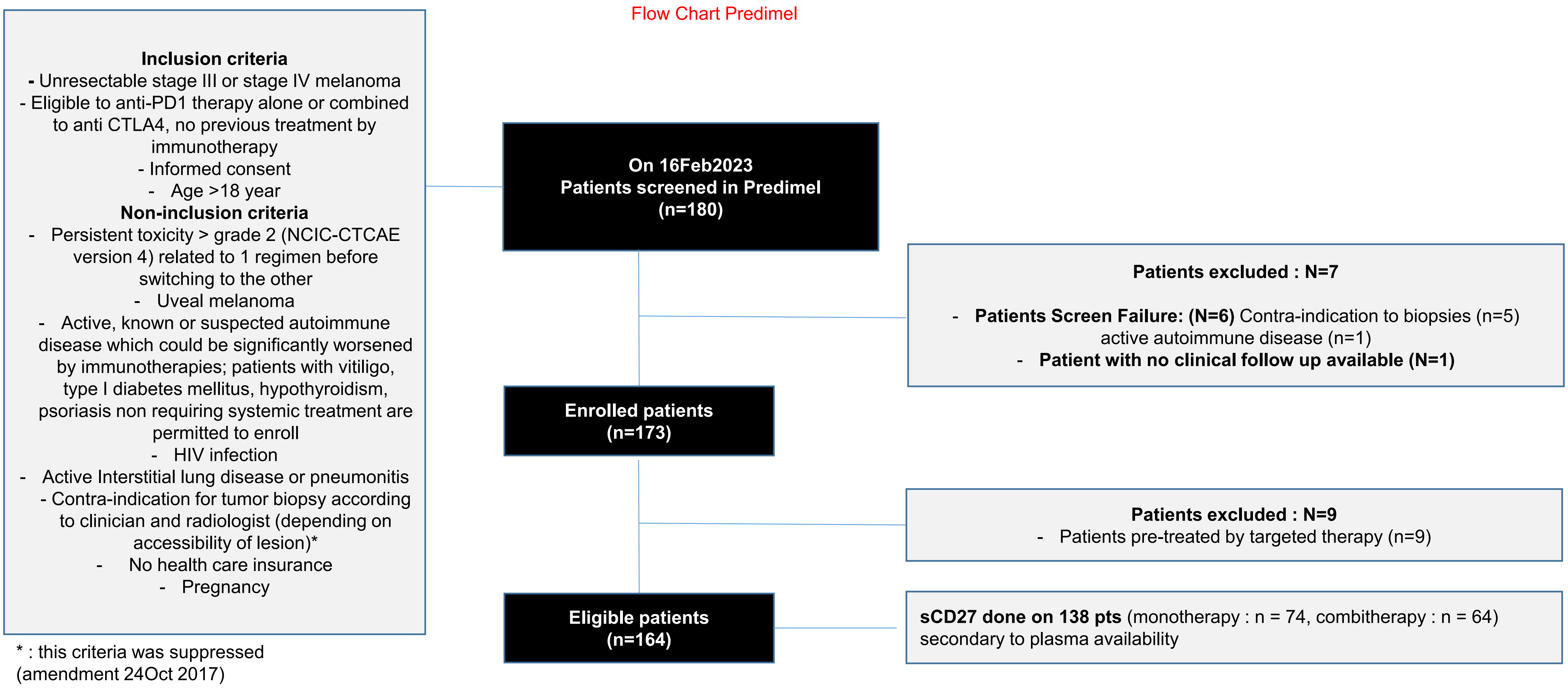

Appendix Fig S1A

Flow Chart MelBase

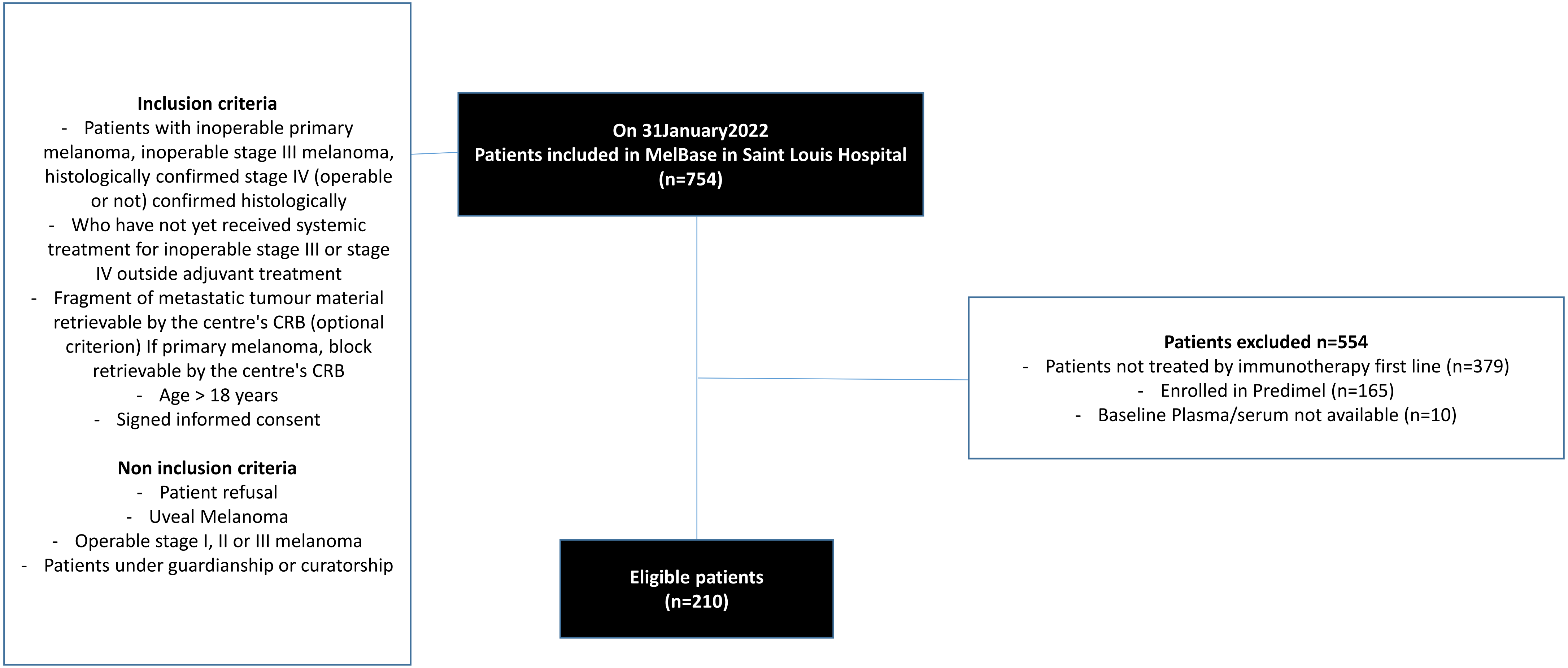

Appendix Fig S1 : Flow chart for the two metastatic melanoma cohorts
A Flow chart for the Predimel cohorts. B : Flow chart for the Melbase cohort

Appendix Fig S1B

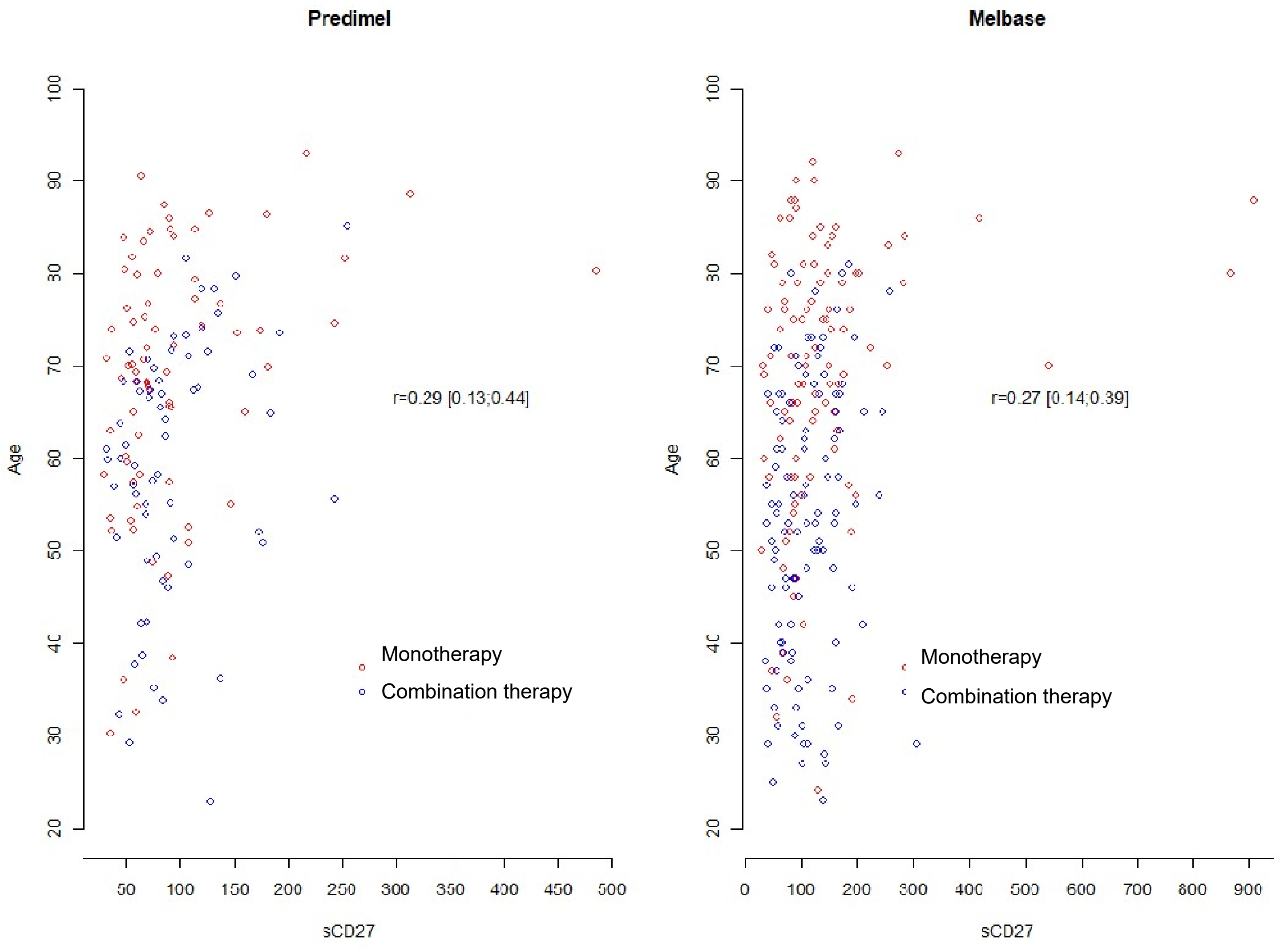

Appendix Fig S2: Correlation between plasma sCD27 concentrations and age of patients

Plasma sCD27 concentrations were plotted against the age of patients at inclusion (years) in the Predimel (left) or Melbase (right) cohorts. r=Spearman correlation coefficient (coefficient is shown on each panel)

Parameters	Values	N	Statistics	N	Statistics	N	Statistics	p-value
		164	All	82	Monotherapy	82	Combination	
**Age, years, median**		164	67·3 [56·1-74·45] (22·8-93)	82	70·75 [60·78-79·97] (30·2-93)	82	60·9 [52·35-69·92] (22·8-85·1)	<0·0001
**Sex**	Male	107	65%	53	65%	54	66%	1·00
	Female	57	35%	29	35%	28	34%	
	NA	10		3		7		
**Braf mutation**	No	141	86%	73	89%	68	83%	0·37
	Yes	23	14%	9	11%	14	17%	
**Nras mutation**	No	135	82%	65	79%	70	85%	0·41
	Yes	29	18%	17	21%	12	15%	
**Brain metastases**	No	154	94%	77	94%	77	94%	1·00
	Yes	10	6%	5	6%	5	6%	
**Liver metastases**	No	142	87%	79	96%	63	77%	0·0004
	Yes	22	13%	3	4%	19	23%	
**More than 3 metastatic sites**	No	147	90%	77	94%	70	85%	0·12
	Yes	17	10%	5	6%	12	15%	
**ECOG PS**	>= 2	43	29%	25	33%	18	25%	0·37
	0 or 1	104	71%	51	67%	53	75%	
	NA	17		6		11		
**LDH**		153	2·75 [1·93-4·17] (1·21-23·04)	78	2·675 [1·915-4·005] (1·21-22·65)	75	2·85 [1·93-4·475] (1·44-23·04)	0·44
**LDH > Normal values**	No	112	73%	63	81%	49	65%	0·044
	Yes	41	27%	15	19%	26	35%	
	NA	11		4		7		
**Neutrophils/Lymphocytes ratio at inclusion**		153	2·594 [1·818-4·098] (0·2493-11·57)	75	2·5 [1·856-3·496] (0·2493-9·837)	78	2·767 [1·807-4·561] (0·2961-11·57)	0·27
**AJJCC M1c at inclusion**	No	98	60%	59	72%	39	48%	0·002
	Yes	66	40%	23	28%	43	52%	
**TMB**		72	6.269 [1.6 ;13.41] (0.0827 ;189.2)	45	5.988 [2.812 ;12.98] (0.215 ;77.44)	27	7.129 [1.58 ;13.7] (0.0827 ;189.2)	0.84
**TMB>10 mut/Mb**	No	46	64%	31	69%	15	56%	0.31
	Yes	26	36%	14	31%	12	44%	
	NA	92						
**Total CD8 cells/mm2**		70	258.6 [24.23 ;1243] (0 ;15020)	47	351.9 [31.64 ;1121] (0 ;15020)	23	200 [15.78 ;2280] (1.516 ;7600)	0.87
**CRP**		138	4.195 [1.292 ;16.26] (1 ;338.8)	74	3.46 [1.09 ;9.88] (1 ;338.8)	64	5.935 [2.025 ;24.95] (1 ;280)	0.096
**CRP>5 mg/mL**	No	75	54%	45	61%	30	47%	0.12
	Yes	63	46%	29	39%	34	53%	
	NA	26		8		18		
**IL-6 >10 pg/mL**	No	119	86%	66	89%	53	83%	0.33
	Yes	19	14%	8	11%	11	17%	
	NA	26		8		18		

Appendix Table S1. Characteristics of patients treated with monotherapy or combination therapy in the PREDIMEL cohort.

The distribution of biological and clinical variables considered as a continuous (age, neutrophil/lymphocyte ratio, LDH, TMB, Total CD8+cells/mm2) or dichotomized (sex, BRAF mutation, Nras mutation, cerebral metastasis, more than 3 metastatic sites, ECOG PS, LDH > normal values, AJCC M1c, TMB>10mut/Mb, CRP>5mg/mL, IL-6>10pg/mL) at inclusion in the PREDIMEL cohort in patients treated either with monotherapy or combination therapy is shown. The imbalance in this distribution

between patients treated with monotherapy or combination therapy was tested using the Wilcoxon's rank sum test for continuous variables and Fisher's exact test for categorical variables. A probability value of $p<0{\cdot}05$ was considered significant. Data are presented as median, (min-max range) and [Interquartile range IQR] or percentage.

Parameters	Value	N	Statistics	N	Statistics	N	Statistics	p-value
		210	Total	102	monotherapy	108	Combination	
Age, years [median]		210	63 [50-73] (23-93)	102	70·5 [60-80] (24-93)	108	54 [41·5-65·25] (23-81)	<0·0001
Sex	Male	126	60%	63	61·8 %	63	58·3 %	0·67
	Female	84	40%	39	38·2 %	45	41·7 %	
Braf mutation	No	146	69·9 %	84	82·4 %	62	57·9 %	0·0001
	Yes	63	30·1 %	18	17·6 %	45	42·1 %	
	NA	1		0		1		
Nras mutation	No	115	62·5 %	55	58·5 %	60	66·7 %	0·29
	Yes	69	37·5 %	39	41·5 %	30	33·3 %	
	NA	26		8		18		
Brain metastasis	No	172	83·5 %	86	86%	86	81·1 %	0·45
	Yes	34	16·5 %	14	14%	20	18·9 %	
	NA	4		2		2		
Liver metastasis	No	163	79·1 %	84	84%	79	74·5 %	0·12
	Yes	43	20·9 %	16	16%	27	25·5 %	
	NA	4		2		2		
More than 3 metastatic sites	No	170	82·5 %	89	89%	81	76·4 %	0·027
	Yes	36	17·5 %	11	11%	25	23·6 %	
	NA	4		2		2		
ECOG PS	>=2	47	22·4 %	21	20·6 %	26	24·1 %	0·62
	0 or 1	163	77·6 %	81	79·4 %	82	75·9 %	
LDH/100		193	3·26 [2·21-4·03] (1·07-58·68)	94	3·145 [2·125-4·005] (1·21-28·43)	99	3·5 [2·44-4·035] (1·07-58·68)	0·19
LDH > normal values	No	154	79·8 %	76	80·9 %	78	78·8 %	0·86
	Yes	39	20·2 %	18	19·1 %	21	21·2 %	
	NA	17		8		9		
Neutrophils/Lymphocytes ratio at inclusion		202	2·61 [1·861-3·771] (0·03677-36·19)	98	2·59 [1·76-3·51] (0·036-36·19)	104	2·656 [1·963-3·847] (0·2961-20·37)	0·61
AJCC M1 at inclusion	No	111	52·9 %	63	61·8 %	48	44·4 %	0·013
	Yes	99	47·1 %	39	38·2 %	60	55·6 %	
CRP		197	3.58 [1.09 ;14.57] (1 ;228)	97	3.37 [1.18 ;9.55] (1 ;194.1)	100	3.675 [1.08 ;17.07] (1 ;228)	0.59
CRP>5 mg/mL	No	114	58%	59	61%	55	55%	0.47
	Yes	83	42%	38	39%	45	45%	
	NA	13		5				
IL-6>10 pg/mL	No	178	91%	87	90%	91	92%	0.59
	Yes	18	9%	10	10%	8	8%	
	NA	14		5		9		

Appendix Table S2: Characteristics of patients treated with monotherapy or combination therapy in the MelBase cohort.

The distribution of biological and clinical variables considered as a continuous (age, neutrophil/lymphocyte ratio, LDH, CRP) or dichotomized (sex, BRAF mutation, NRAS mutation, cerebral metastasis, more than 3 metastatic sites, ECOG PS, LDH > normal values, AJCC M1c, CRP>5mg/mL, IL-6>10pg/mL) at inclusion in the MelBase cohorts in patients treated either with monotherapy or

combination therapy is shown. The imbalance in this distribution between patients treated with monotherapy or combination therapy was assessed using the Wilcoxon's rank sum test for continuous variables and Fisher's exact test for categorical variables. A probability value of $p < 0{\cdot}05$ was considered significant. Data are presented as median, (min-max range) and [Interquartile range IQR] or percentage.

PREDIMEL PFS

Variable	Values	N	Nevent	HR	95%CI	P value
Breslow at diagnosis		82	46	0.98	0.91-1.06	0.57
Ulceration	No	34	19	1		
	Yes	40	22	1.14	0.61-2.11	0.68
	Unknown	8	5	1.46	0.54-3.94	0.46
Lymph nodes	N0	60	33	1		
	N+	22	13	1.12	0.59-2.13	0.73
Localization	Mucosae/Palms/Soles/Nails	7	5	1		
	Other skin	75	41	0.72	0.28-1.82	0.48
Histology	Other	77	44	1		
	Mucosal-acral lentiginous	5	2	0.71	0.17-2.93	0.64

PREDIMEL OS

Variable	Values	N	Nevent	HR	95%CI	P value
Breslow at diagnosis		82	8	0.97	0.80-1.18	0.79
Ulceration	No	34	3	1		
	Yes	40	4	1.37	0.31-6.14	0.68
	Unknown	8	1	1.94	0.20-18.9	0.57
Lymph nodes	N0	60	4	1		
	N+	22	4	2.76	0.69-11.1	0.15
Localization	Mucosae/Palms/Soles/Nails	7	1	1		
	Other skin	75	7	0.67	0.08-5.46	0.71
Histology	Other	77	8			
	Mucosal-acral lentiginous	5	0	n/a	n/a	0.50*

* p-value of log-rank test, due to absence of death events in one group

Appendix Table S3: **Clinical variables at diagnosis from PREDIMEL in the prediction of progression-free survival (PFS) and overall survival (OS) and in melanoma patients treated by anti-PD-1 alone**. Forest plot displaying the univariate Cox's model Hazard Ratios (HRs) for PFS (Top) and OS (Bottom) and 95% confidence intervals (CI) of baseline clinical variables in the PREDIMEL cohort. Concentration of sCD27 was evaluated either as a continuous variable or dichotomized using a 100U/ml cut-off. A two-sided $p<0.05$ was considered significant. *N* indicates the number of patients in the subgroup defined by the characteristics and *N event* indicates for each subgroup the number of events among them.

Antibody information for multiplex IHC panel			
Primary Ab	Concentration of primary Ab (µg/ml)	Secondary Ab	TSA-Dye
CD70 (R&D) MAB2738	1	Anti-Mouse ImmunoReagent GAMHRP-050	CF594 Biotium 92174
CD8 (CST) 70306S	0.1	Anti-Mouse ImmunoReagent GAMHRP-050	CF680R Biotium 92196
CD27 (Abcam) AB131254	0.15	Anti-Rabbit ImmuoReagent GARHRP-050	CF430 Biotium 96053
Melan-A (NovusBio) NBP1-30151 (clone A19-P)	0.7	Anti-Rabbit ImmuoReagent GARHRP-050	CF555 Biotium 92214

Appendix Table S4. Antibody information for multiplex Immunofluorescence panel
