# Supplementary figures and images for: Soluble CD27 differentially predicts resistance to anti-PD1 alone but not with anti-CTLA-4 in melanoma
Source: EMBO Mol Med. 2025 Mar 27;17(5):909–22. doi: 10.1038/s44321-025-00203-9 (PMC12081602; doi:10.1038/s44321-025-00203-9)

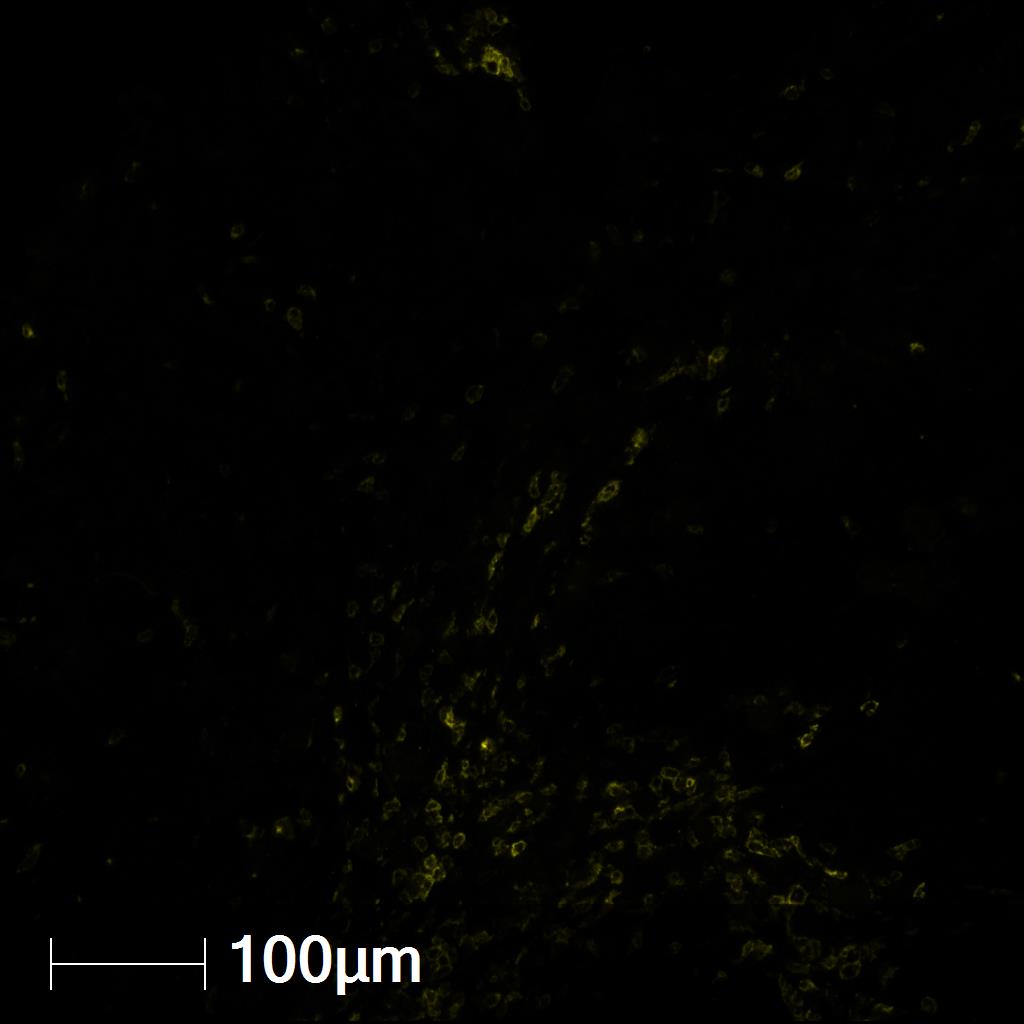

Supplement: Supplementary file 7 — Source data Fig. 1 [file 44321_2025_203_MOESM7_ESM.zip › Figure 1/Fig1A/Fig 1A/CD27.jpg]

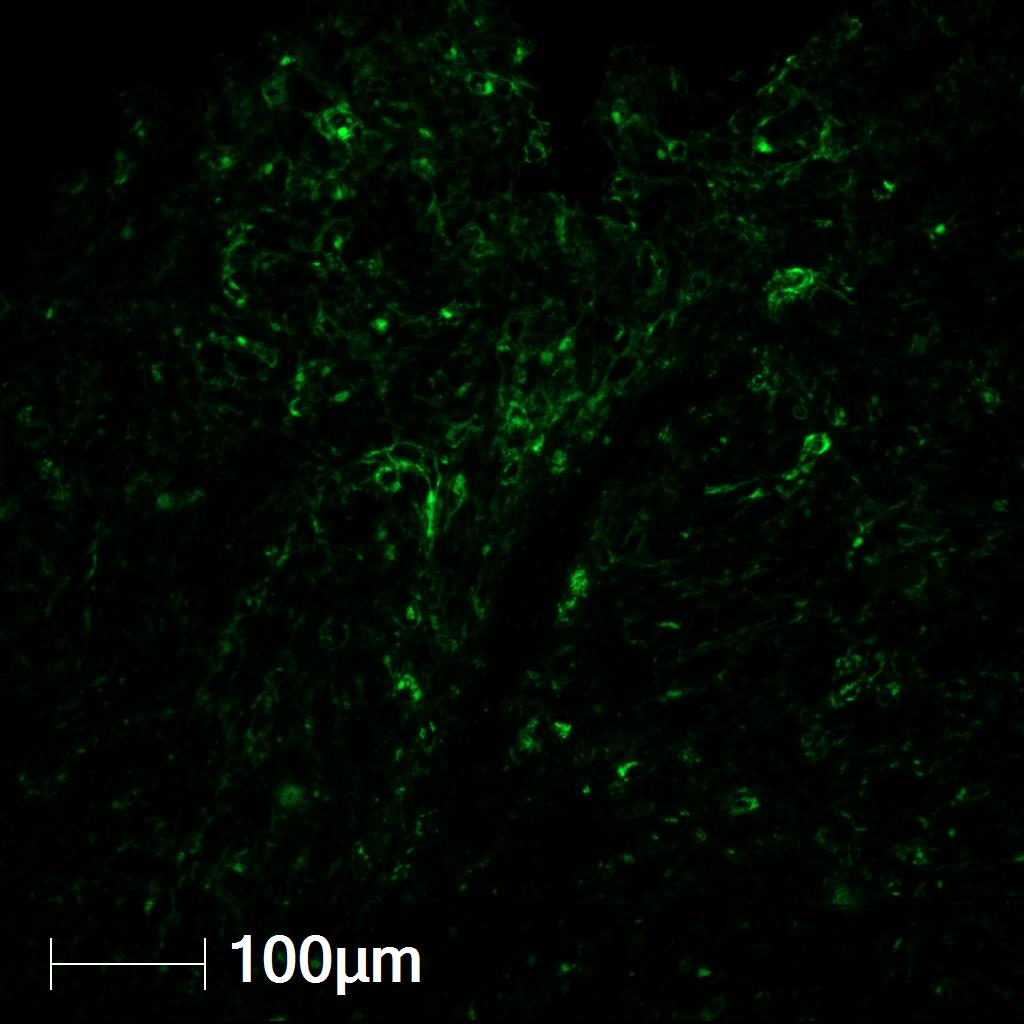

Supplement: Supplementary file 7 — Source data Fig. 1 [file 44321_2025_203_MOESM7_ESM.zip › Figure 1/Fig1A/Fig 1A/CD70.jpg]

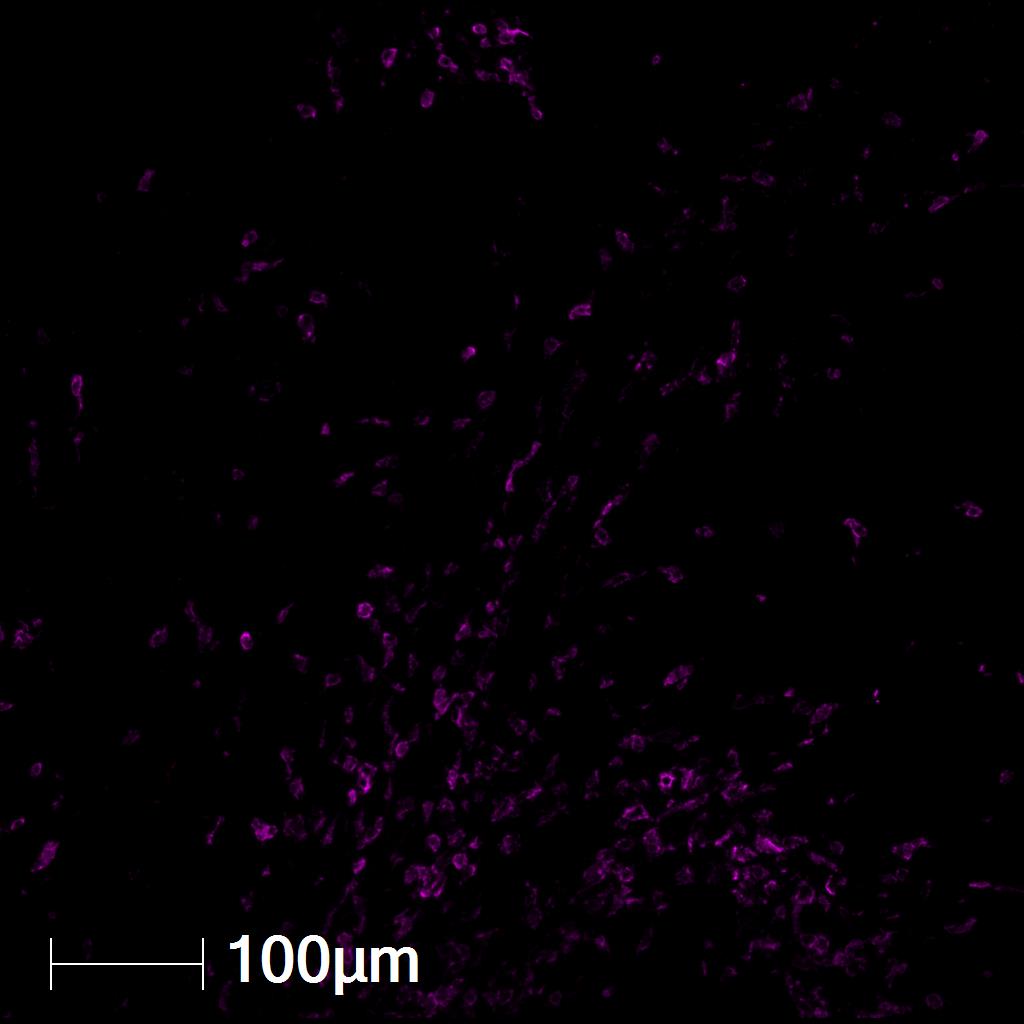

Supplement: Supplementary file 7 — Source data Fig. 1 [file 44321_2025_203_MOESM7_ESM.zip › Figure 1/Fig1A/Fig 1A/CD8.jpg]

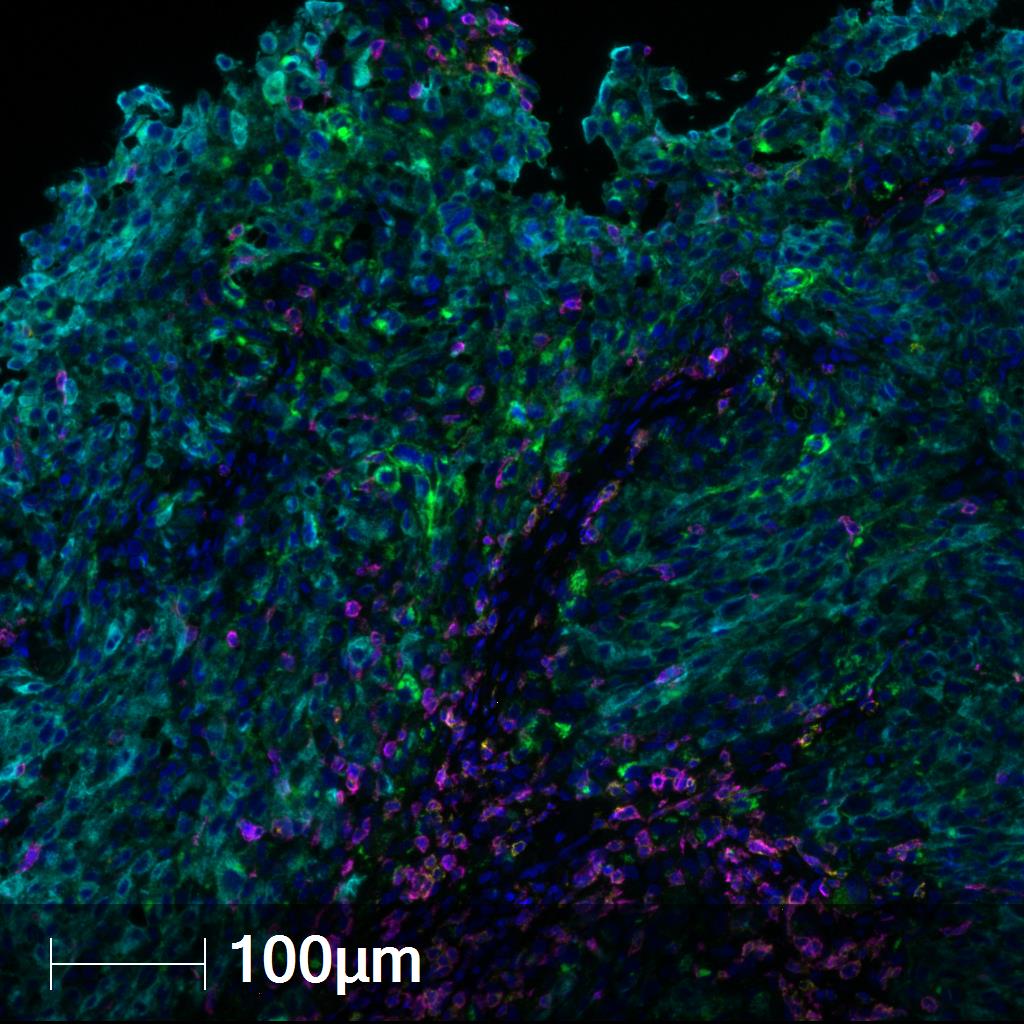

Supplement: Supplementary file 7 — Source data Fig. 1 [file 44321_2025_203_MOESM7_ESM.zip › Figure 1/Fig1A/Fig 1A/component.jpg]

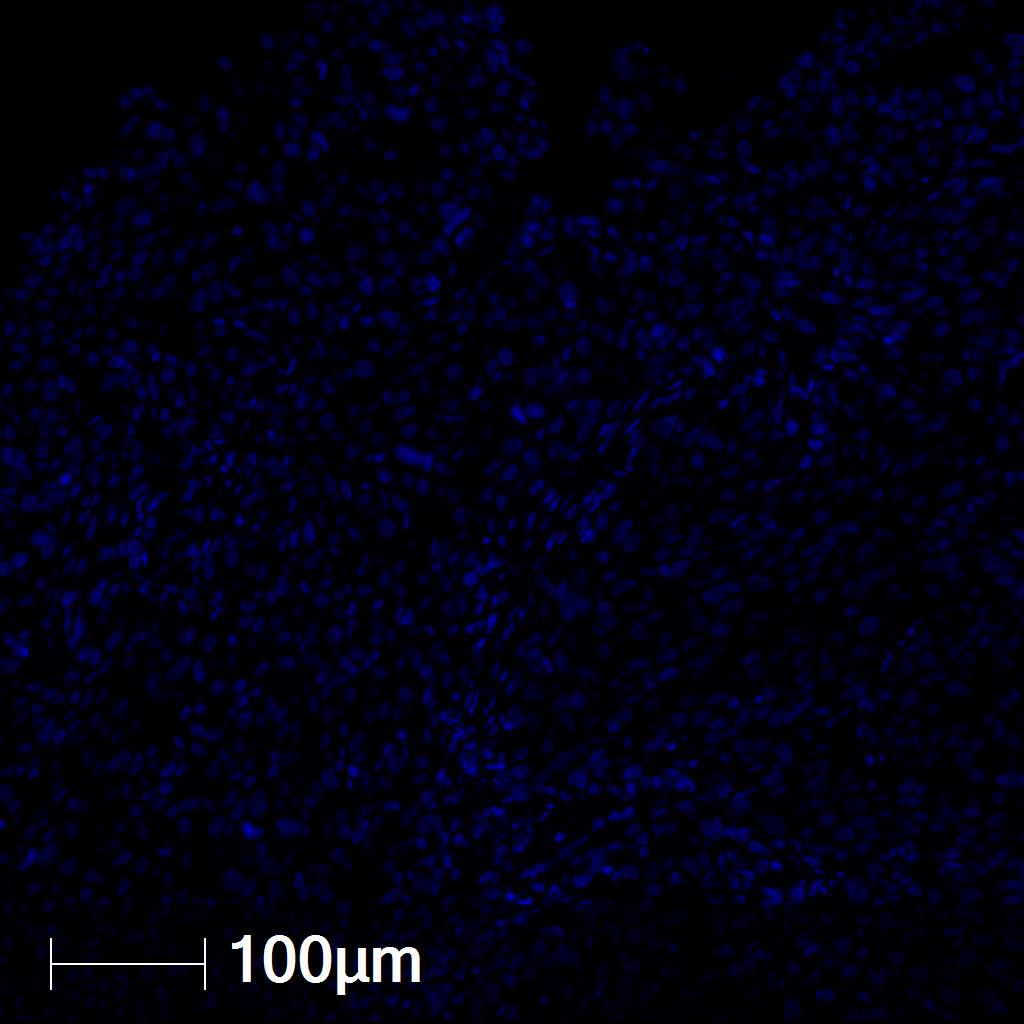

Supplement: Supplementary file 7 — Source data Fig. 1 [file 44321_2025_203_MOESM7_ESM.zip › Figure 1/Fig1A/Fig 1A/DAPI.jpg]

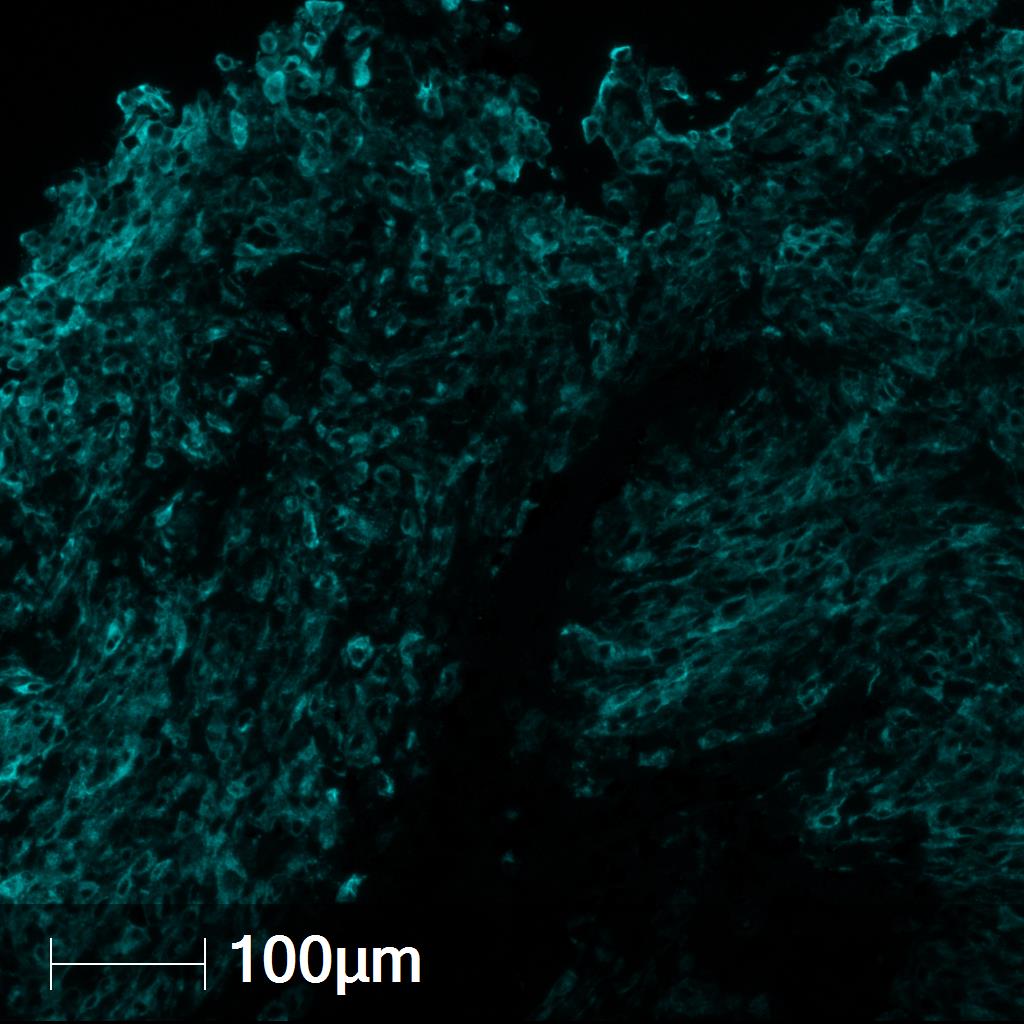

Supplement: Supplementary file 7 — Source data Fig. 1 [file 44321_2025_203_MOESM7_ESM.zip › Figure 1/Fig1A/Fig 1A/melan-A.jpg]

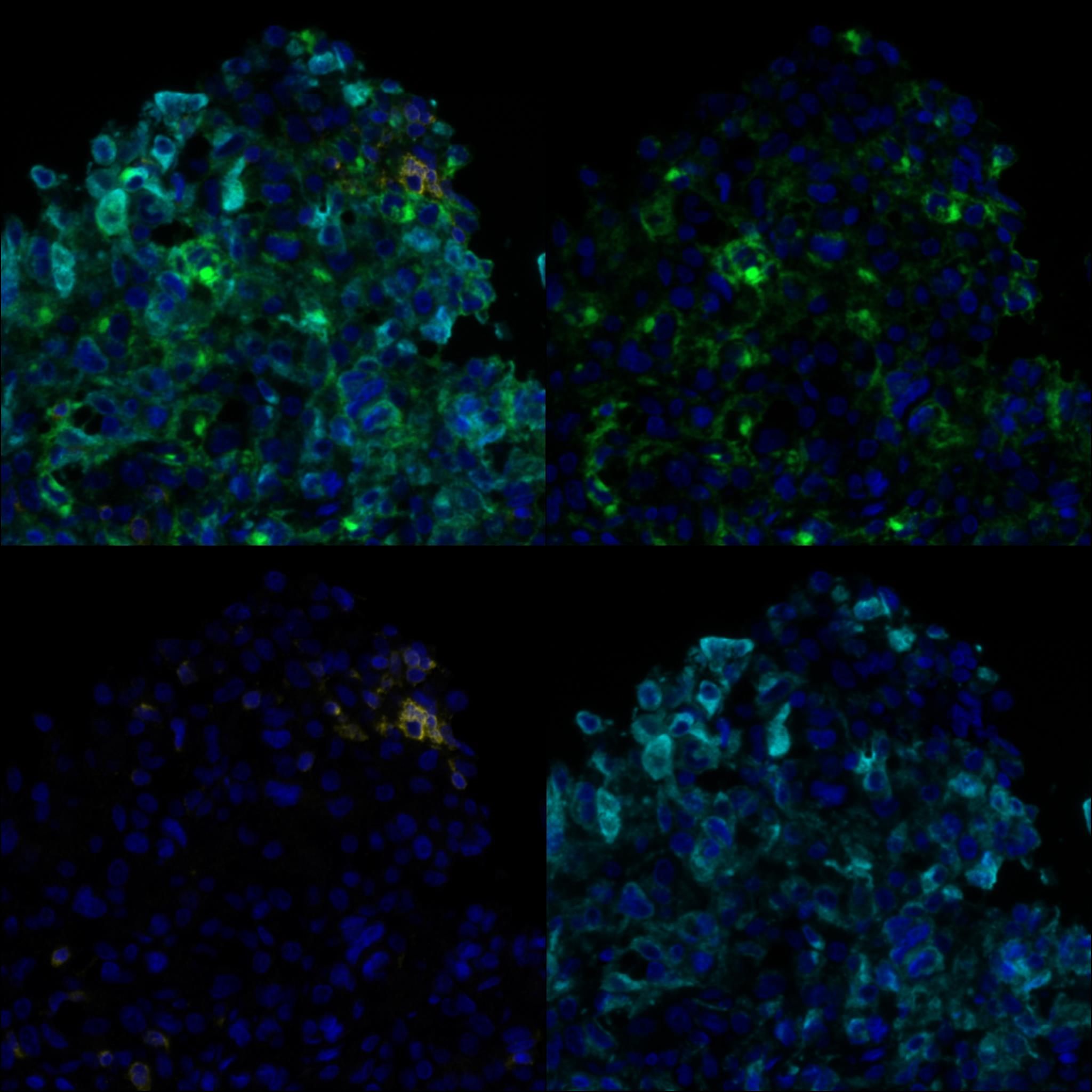

Supplement: Supplementary file 8 — Source data Fig. 2 [file 44321_2025_203_MOESM8_ESM.zip › Figure 2/Fig2A/Representative image.jpg]

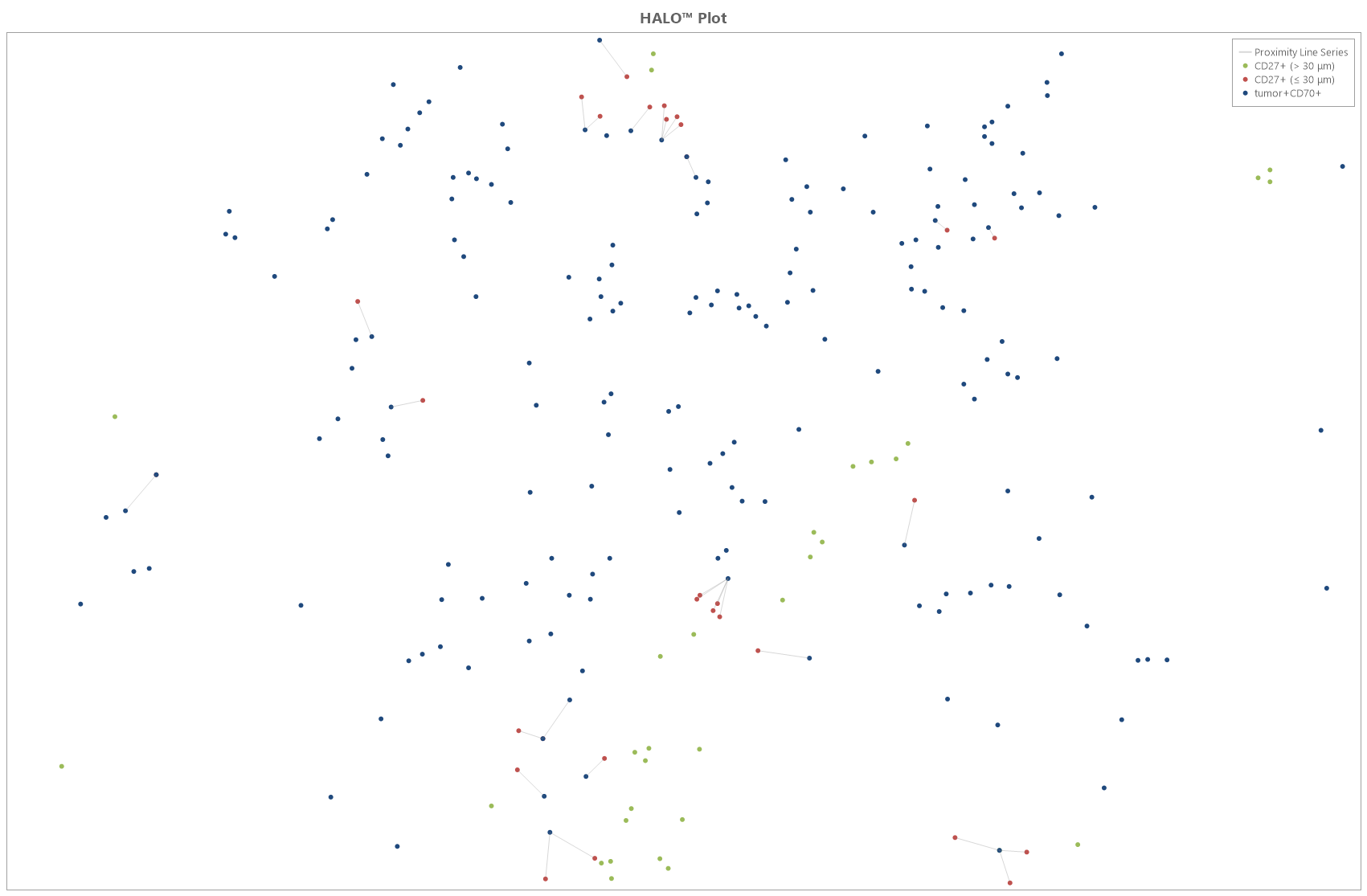

Supplement: Supplementary file 8 — Source data Fig. 2 [file 44321_2025_203_MOESM8_ESM.zip › Figure 2/Fig2A/Spatial plot.png]

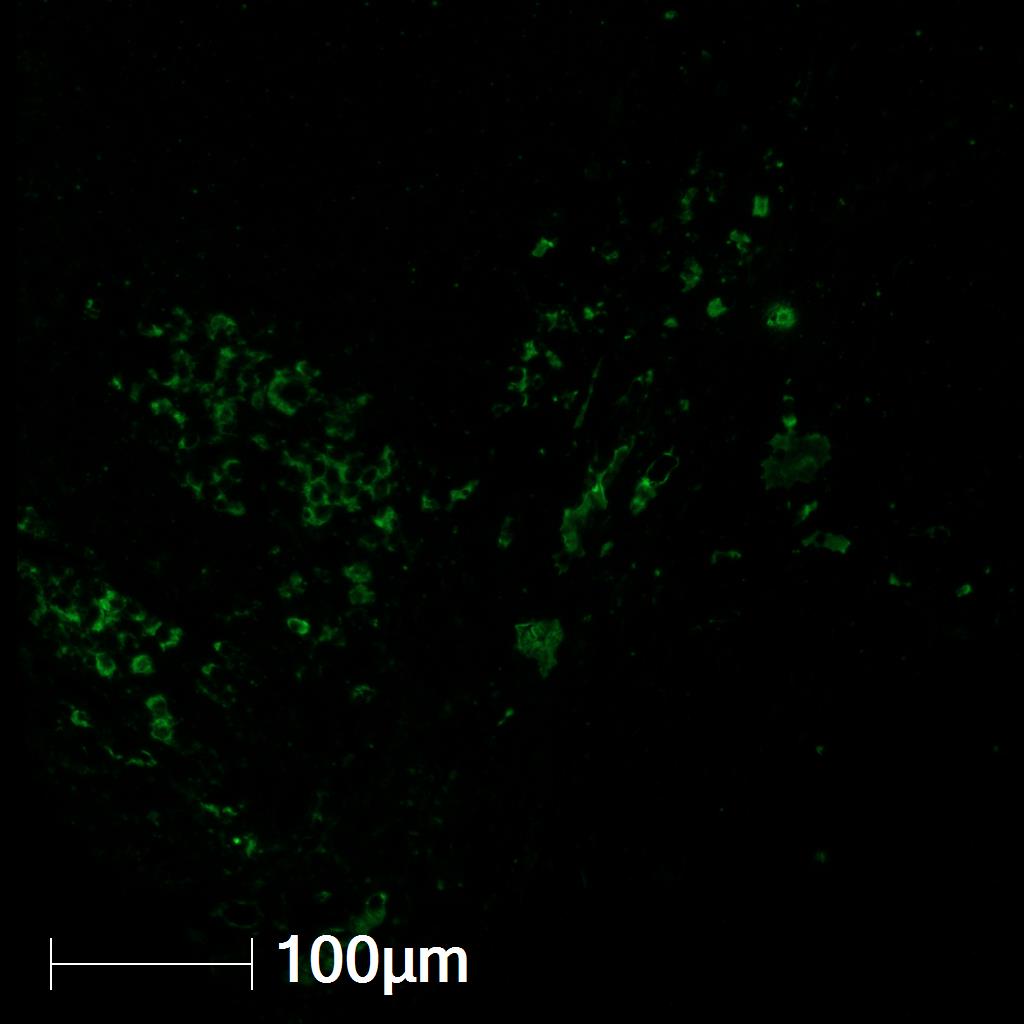

Supplement: Supplementary file 8 — Source data Fig. 2 [file 44321_2025_203_MOESM8_ESM.zip › Figure 2/Fig2B/01-060 CD70.jpg]

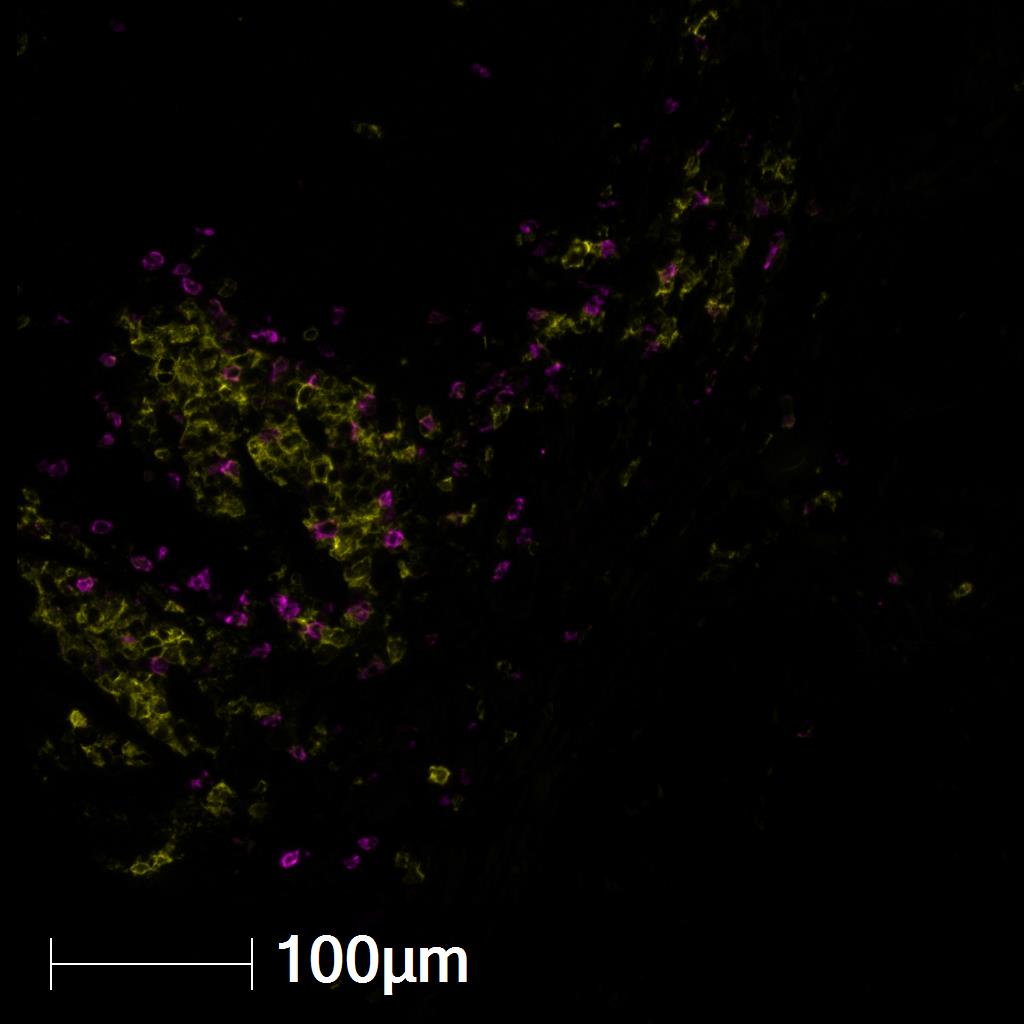

Supplement: Supplementary file 8 — Source data Fig. 2 [file 44321_2025_203_MOESM8_ESM.zip › Figure 2/Fig2B/01-060 CD8+CD27+.jpg]

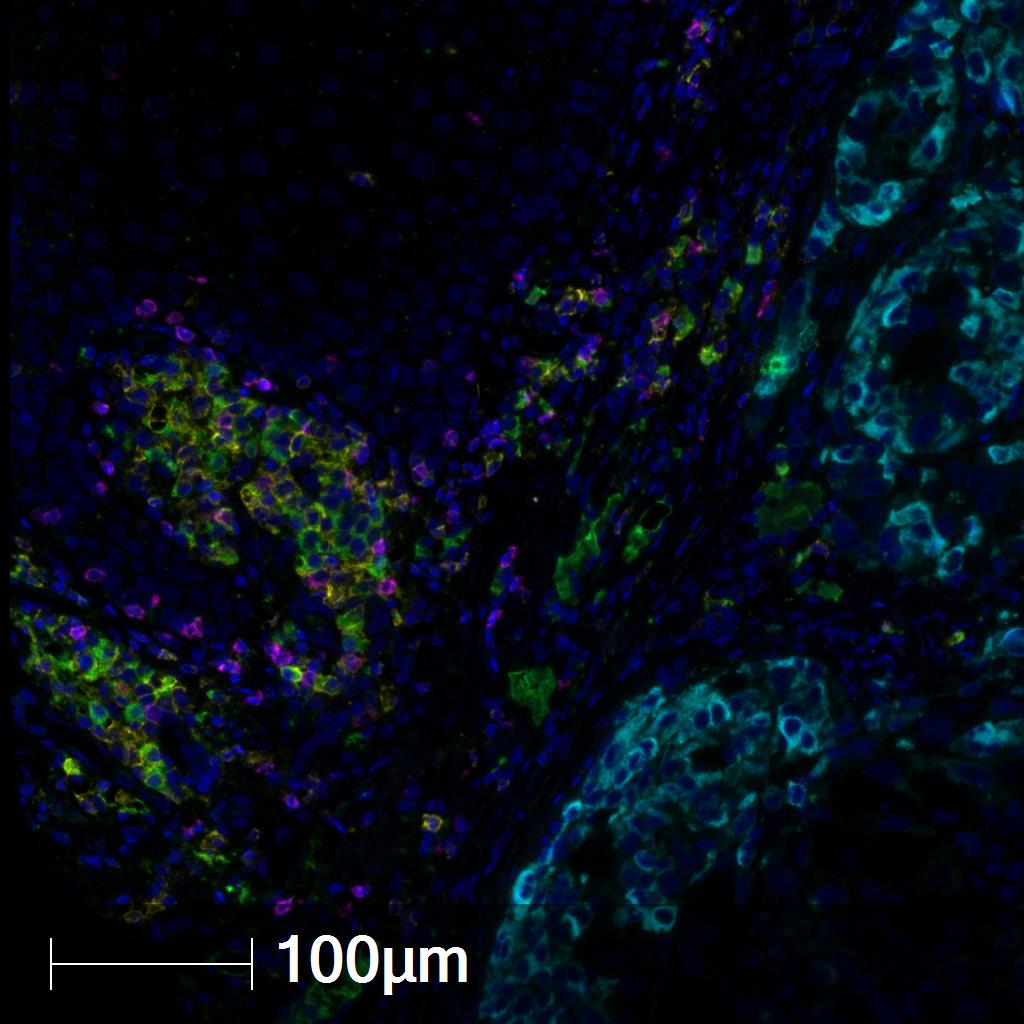

Supplement: Supplementary file 8 — Source data Fig. 2 [file 44321_2025_203_MOESM8_ESM.zip › Figure 2/Fig2B/01-060 component.jpg]

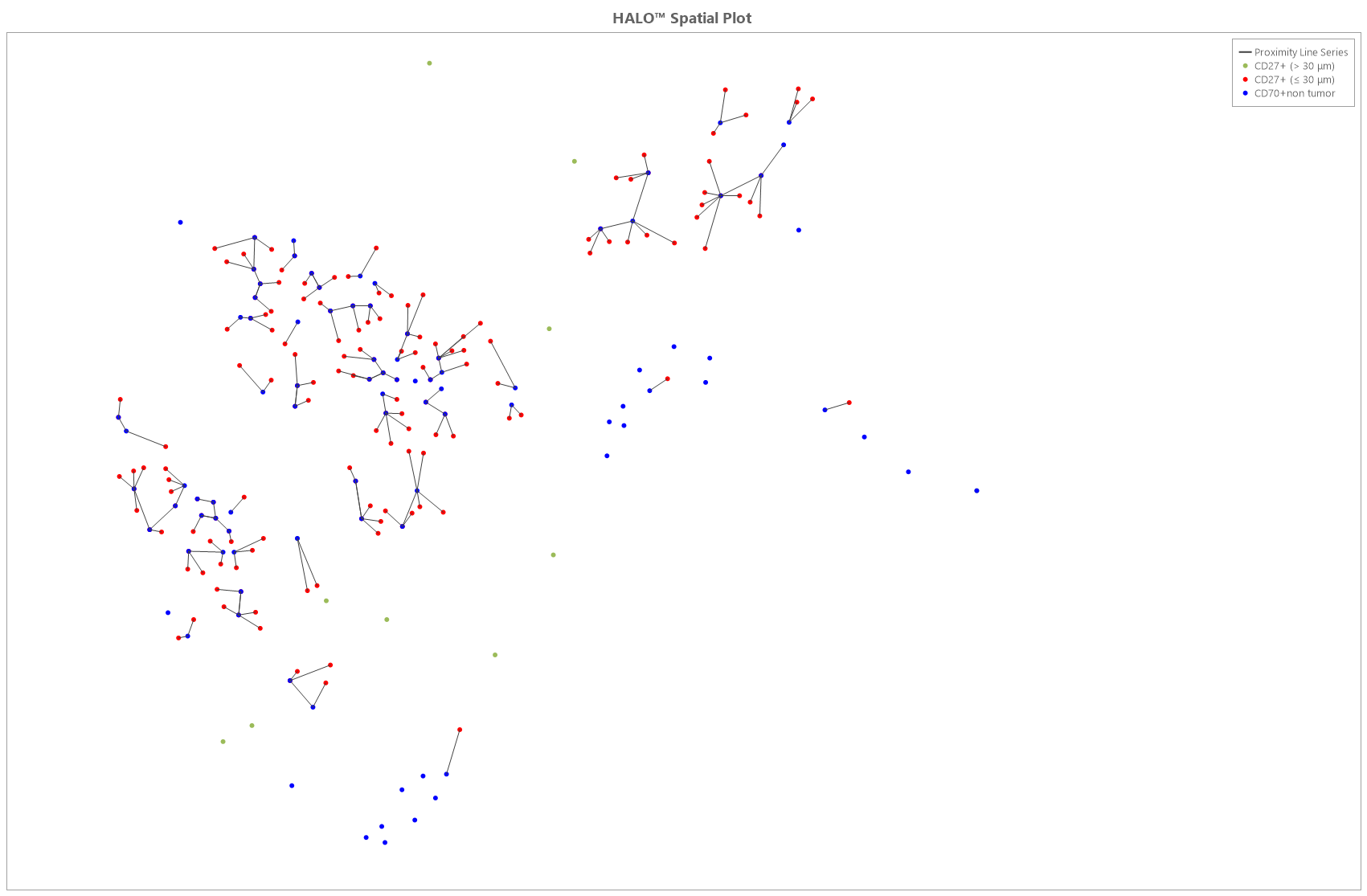

Supplement: Supplementary file 8 — Source data Fig. 2 [file 44321_2025_203_MOESM8_ESM.zip › Figure 2/Fig2B/Spatial plot.png]
